# Future of the Genetic Code

**DOI:** 10.3390/life7010010

**Published:** 2017-02-28

**Authors:** Hong Xue, J. Tze-Fei Wong

**Affiliations:** Division of Life Science and Applied Genomics Center, Hong Kong University of Science & Technology, Clear Water Bay, Hong Kong, China; hxue@ust.hk

**Keywords:** synthetic life, non-canonical amino acid, rewritten genetic code, restrictive post-transcriptional modification, anticodon identity element

## Abstract

The methods for establishing synthetic lifeforms with rewritten genetic codes comprising non-canonical amino acids (NCAA) in addition to canonical amino acids (CAA) include proteome-wide replacement of CAA, insertion through suppression of nonsense codon, and insertion via the pyrrolysine and selenocysteine pathways. Proteome-wide reassignments of nonsense codons and sense codons are also under development. These methods enable the application of NCAAs to enrich both fundamental and applied aspects of protein chemistry and biology. Sense codon reassignment to NCAA could incur problems arising from the usage of anticodons as identity elements on tRNA, and possible misreading of NNY codons by UNN anticodons. Evidence suggests that the problem of anticodons as identity elements can be diminished or resolved through removal from the tRNA of all identity elements besides the anticodon, and the problem of misreading of NNY codons by UNN anticodon can be resolved by the retirement of both the UNN anticodon and its complementary NNA codon from the proteome in the event that a restrictive post-transcriptional modification of the UNN anticodon by host enzymes to prevent the misreading cannot be obtained.

## 1. Introduction

The 100th anniversary of the birth of Francis Crick is an occasion that calls for celebration of the double helix, triplet genetic code, tRNA, wobble rules, and aspects of molecular biology and neurobiology advanced by his gifted insights.

The cooperation between RNA and protein founded on the triplet genetic code is pivotal to life, and the development of the standard genetic code is the centerpiece in life’s emergence. The sharing of the same protein alphabet by all living species suggests that the alphabet determined by the standard code predated the earliest divergence of organisms. Yet evidence suggests that the early code began with prebiotically-derived amino acids and expanded to include biosynthetically derived amino acids, thus predicting that the code is intrinsically a mutable code. To put this prediction to the test, experiments were performed in 1983 to determine the code’s mutability. These experiments led to the isolation of genetic code mutants of the Trp-auxotroph *Bacillus subtilis* QB928 in which Trp has been either replaced in the code by its normally toxic fluoro analogues 4FTrp (4-fluoroTrp), 5FTrp, and 6FTrp; or even displaced entirely by 4FTrp to become an inhibitory analogue [1,2,3,4,5]. This proof of the mutability of the code opens up the code to revision and expansion, encoding NCAAs alongside CAAs in the protein alphabet.

## 2. Synthetic Lifeform Production

Since the genetic code is the most basic attribute of living systems, genetic code mutants represent the ultimate test-tube evolution [6]. Accordingly, organisms such as the *B. subtilis* strains that have rejected Trp from their genetic codes may be designated as synthetic lifeforms, distinct from synthetic biological constructs that contain novel genes and gene ensembles, but adhere strictly to the universal protein and nucleic acid alphabets [7]. The synthetic lifeforms with altered protein alphabets can be either optional or mandatory in their utilization of NCAAs, and the NCAAs can be incorporated proteome-wide or localized to specific protein sites. Synthetic lifeforms using a revised DNA alphabet where thymine is replaced by 5-chlorouracil on an optional or mandatory basis [8,9,10], or an extra unnatural base pair exemplified by dNaM-d5SICS has been added to the A-T and G-C pairs [11,12,13], have since been isolated as well.

Since the isolation of the first synthetic lifeforms from *Bacillus subtilis*, a number of different methods have been developed for the production of synthetic lifeforms equipped with mutated genetic codes and altered protein alphabets:

*Proteome-wide replacement of CAA by NCAA*: NCAAs that have acquired encoding by the genetic code include fluoroTrps in *B. subtilis* [1,4], *E. coli* and coliphages [14,15,16], and l-β-(thieno[3,2-b]-pyrrolyl)alanine in *E. coli* [17]. With respect to 4FTrp, genome sequencing of the successive mutant strains leading from wild-type *B. subtilis* to the HR23 strain which rejects Trp from its genetic code, and on to revertant TR7 strains where Trp rejoins the code, revealed how 4FTrp and Trp could be admitted into or excluded from the code as the result of a limited number of mutations in oligogenic-barrier genes that have preserved the protein alphabet against change through the ages [2,4,5].

*NCAA insertion through suppression of a nonsense codon*: To safeguard the fidelity of translation, aminoacyl-tRNA synthetases (ARSs) react with tRNAs cognate to the amino acid substrate, but not tRNAs cognate to other amino acids. Surprisingly, however, when *E. coli* ARSs were presented with tRNAs from other species, they reacted well with most tRNAs sourced from other bacteria, but poorly with numerous tRNAs from another biological domain: with *Halobacterium cutirubrum* tRNAs, they displayed only 1%–3% reactivity with tRNAs for Phe, Asp, Lys, and Pro, 0.4%–0.5% with tRNAs for Tyr, Leu, and Arg, and 0% with tRNAs for Ser [18,19]. Such strikingly low inter-domain reactivities made possible the design of ‘orthogonal’ ARS-tRNA pairs that do not interact productively with tRNAs and ARSs of the host cell [20], notably based on the archaeal *Methanocaldococcus* TyrRS, *Methanosarcina* PylRS, and SepRS from methanogens [21]. Using this approach, a wide range of site-specific NCAA incorporations in bacterial and eukaryotic hosts including *C. elegans* and *Xenopus* oocytes have been achieved based on suppression of nonsense codon [22,23,24,25,26]. Limitations of NCAA insertion through nonsense codon suppression include low efficiency of NCAA translation at levels close to those displayed by near-cognate CAAs, and competition from release factor for the nonsense codon [21]. These limitations could be overcome by directed evolution, with up to three-fold reduction of promiscuous aminoacylation of orthogonal tRNA by endogenous ARS through a combination of positive selection for amber suppression activity and negative selection toward ARS [27,28]. Efficiency also may be enhanced by the use of quadruplet-decoding ribosomes [29].

*NCAA encoding through pyrrolysine and selenocysteine pathways*: Since the nonsense codon UAG can perform double duty as a termination signal and a codon for pyrrolysine (Pyl) depending on the sequence context, PylRS and its cognate tRNA(Pyl) represent an endogenous orthogonal ARS-tRNA pair [30], and NCAA encoding has been achieved through their adaptation to NCAAs [31,32,33]. Although PylRS is comparable in catalytic efficiency to other ARSs with regard to Pyl-tRNA(Pyl) formation, engineered PylRS displayed 1000-fold reduction in catalytic efficiency toward NCAA [21], again indicating the need for enhancement. The nonsense codon UGA, likewise, can perform double duty as a termination signal and a codon for selenocysteine (Sec). The adaptation of the Sec pathway to NCAA encoding is rendered difficult by the requirement for a selenocysteine insertion sequence (SECIS) motif, and the non-binding of Sec-tRNA(Sec) to EF-Tu. However, both of these hurdles have been removed through the development of an effective tRNA(Sec) that incorporated Sec into proteins via EF-Tu binding, thus converting the highly restricted occurrence of Sec only at SECIS-directed mRNA contexts to unconstrained insertion as an NCAA anywhere in the proteome [34,35]. This liberated Sec pathway therefore can be adapted to the incorporation of NCAAs as in the case of the Pyl pathway.

*Proteome-wide reassignment of nonsense codon*: To overcome the restriction of site-specific incorporation of NCAA, nonsense codon encoding of NCAAs is being extended to the proteome-wide scale by replacing all 314 UAG nonsense codons in *E. coli* by the synonymous UAA nonsense codon using multiplex automated genome engineering (MAGE) followed by hierarchical conjugative assembly genome engineering (CAGE). The retired UAG codon is thus ready for proteome-wide reassignment to NCAA [36].

*Proteome-wide reassignment of sense codon*: The genetic code contains only three nonsense codons but 61 sense codons that might be reassignable to NCAAs. Reserving two codons for each CAA other than Met and Trp still leaves many sense codons for reassignment to NCAAs. Reassignments of the rare AGG and CGG codons of Arg to NCAAs have been achieved for homoArg and N6-(1-iminoethyl)Lys via the pyrrolysine pathway [37,38,39]. As well, genome-wide replacement of 13 rare sense codons from a panel of 42 highly-expressed essential genes of *E. coli* by their synonymous codons was functionally tolerable [40], and an orthogonal IleRS-tRNA(Ile) pair from *Mycoplasma mobile* was found that could decipher the AUA sense codon of *E. coli*, indicating the feasibility of reassigning this codon to NCAA [41]. General reassignment of non-rare codons on a proteome-wide basis will entail large scale genome reengineering employing either genome editing procedures or synthetic genomes exemplified by the *Mycoplasma mycoides* JCV1-syn1.0 genome [42]. To cope with the possibly severe adverse effects of such reassignment on the overall performance of the proteome, it might be necessary in some cases to increase gradually the NCAA/CAA incorporation ratio at the codon positions involved instead of an abrupt switch from CAA to NCAA. In addition, important issues that need to be resolved include the usage of anticodon as an identity element on tRNA, and the inherent ability of the UNN anticodon to read all four codons in a family codon box.

## 3. Anticodons as Identity Elements

The selection of tRNA substrate by ARS for aminoacylation is guided by identity elements on tRNA, e.g., the identity elements on *B. subtilis* tRNA(Trp) include G73 and its anticodon as major, and A1-U72, G5-C68, and A9 as minor, identity elements [43]. The discriminator base N73 and the anticodon are the most commonly encountered identity elements [44]. To reassign the CGA and CGG codons of Arg to an NCAA, for instance, these codons need to be replaced by synonymous Arg codons, and the tRNA(Arg)s that read CGA and CGG have to be deleted from the genome, followed by the introduction of orthogonal NcaaARS and tRNA(Ncaa)s bearing UCG anticodon, or UCG and CCG anticodons into the host cell. However, where ArgRS recognizes the anticodon as an identity element, as in *E. coli* and yeast [44], the endogenous ArgRS in the host cell could misread the anticodon on the newly-introduced tRNA(Ncaa)s as its cognate, and proceed to arginylate these tRNA(Ncaa)s [21,38,45]. If the anticodon represents the sole tRNA identity element recognized by ArgRS, the misreading error may be difficult to avoid. On the other hand, if ArgRS recognizes the anticodon along with other co-identity elements, the magnitude of the misreading error would depend on whether the various identity elements act additively or synergistically.

**Figure 1:**
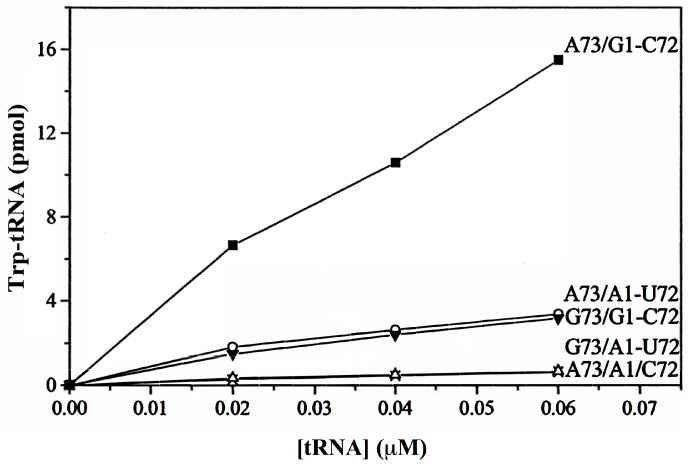
Human TrpRS tryptophanylation of bovine tRNA(Trp) with varied N73 and 1-72 configurations (after [46]).

Earlier, when cloned bovine tRNA(Trp) bearing the A73 and G1-C72 identity elements was reacted with human TrpRS, highly efficient Trp-tRNA(Trp) formation was observed. When either A73 or G1-C72 was altered, efficiency was reduced to ~25%. When both A73 and G1-C72 were altered, or when the 1-72 base pair was disrupted, efficiency was reduced to <2.5% (Figure 1). These findings showed that the identity elements A73 and G1-C72 acted synergistically, rather than additively in attracting Trp-tRNA formation, such that alteration of either A73 or G1-C72 diminished the Trp-tRNA formation by more than half. The physical basis of the synergism between A73 and G1-C72 was demonstrated by NMR spectroscopy: a change in the chemical shift for the G1-C72 base pair became manifest upon replacement of A73 by G73, with the G1 resonance moved in the ^1^H dimension from 11.75 to 12.15 ppm [46].

Moreover, the relative rate of Trp-tRNA formation by *B. subtilis* TrpRS was 100% for *B. subtilis* tRNA, 0.5% for bovine tRNA, and 1.0% for *Archaeglobus fulgidus* tRNA. On the other hand, the relative rate of Trp-tRNA formation by *A. fulgidus* TrpRS was 100% for *A. fulgidus* tRNA, 4% for bovine tRNA and 5% for *B. subtilis* tRNA. Therefore, although the anticodon was a major identity element for *B. subtilis* TrpRS, and both *A. fulgidus* tRNA(Trp) and bovine tRNA(Trp) carried the same CCA anticodon as *B. subtilis* tRNA(Trp), *B. subtilis* TrpRS reacted poorly with bovine and *A. fulgidus* tRNAs, indicating that the presence of a correct anticodon identity element was insufficient to attract substantial Trp-tRNA formation by *B. subtilis* TrpRS [46]. Altogether the findings suggest that the problem of anticodon as identity element could be overcome or diminished in at least some instances by removing from the orthogonal tRNA(Ncaa) all identity elements outside of the anticodon that could attract misacylation by the ARS(Caa) for the CAA donating the codons to the NCAA.

## 4. Misreading by the UNN Anticodon

Since codon boxes are, as a rule, read by less than four anticodons, reading of multiple codons by an anticodon is commonplace. This suggests that reassignment of just a single sense codon to an NCAA can easily trigger misreading of a codon by a non-cognate anticodon belonging to the same box except for specialized instances illustrated by the AUA codon [41]. On the other hand, because Ser is allocated six codons in the code and the anticodon on tRNA(Ser) is also not an identity element in host species such as *E. coli* and yeast [44], reassignment of the entire UCN Ser codon box to an NCAA could be free of both the anticodon identity element problem and the UNN anticodon misreading problem. However, this would leave only the two codons AGU and AGC for Ser. Since there are comparable numbers of Ser and Glu residues in the *E. coli* proteome, and Glu functions well with only two codons, Ser might manage with two as well. In contrast, splitting up an existing 1aa codon box may incur misreading of NNU and NNC codons by the UNN anticodon.

Readings of codons by anticodons are governed by Crick’s wobble rules [47]. Evolution of tRNA sequences indicates that there have been four major stages of wobble development [48]. In Stage 1 wobble, a single tRNA with a UNN anticodon reads all four codons in a codon box. As a result, no codon box could accommodate more than a single amino acid. Stage 2 wobble allocates a GNN-UNN anticodon duo to each box, thus allowing both 1aa and 2aa codon boxes. All eight standard 1aa boxes and five standard 2aa boxes of *Methanopyrus kandleri* (Mka), which is most closely related to the Last Universal Common Ancestor (LUCA), employ Stage 2 wobble. Some archaeons employ in their standard boxes both Stage 2 wobble and Stage 3 wobble which allocates a GNN-UNN-CNN anticodon trio to each box. The majority of *Archaea* species use a Stage 3 wobble for all of their standard boxes. Stage 4 wobble, mainly used by eukaryotes, adds yet another anticodon viz. A(I)NN to its anticodon ensemble.

In Stage 1, the U-1st anticodon reads all four codons of a box in a two-out-of-three reading mode [49,50]. In Stage 2, a G-1st anticodon reads the Y-3rd codons in a box; the U-1st anticodon reads mainly or only the R-3rd codons in a 1aa box, but only the R-3rd codons in a 2aa box. Since the UNN anticodon is inherently capable of two-out-of-three reading, some solution has to be developed to disallow misreading of Y-3rd codons by UNN anticodon in a 2aa box [51,52]. In the course of genetic code evolution, primitive organisms about to acquire Stage 2 wobble had to choose between two solutions to this challenge [53,54]:

Solution I: Prohibit the reading of Y-3rd codons by UNN anticodon through its post-transcriptional modification to form a restricted *UNN anticodon capable of reading the R-3rd but not the Y-3rd codons in a 2aa box; or

Solution II: Abandon the use of the A-3rd codon and U-1st anticodon in the 2aa box altogether, leaving only a C-1st anticodon to read the G-3rd codon and a G-1st anticodon to read the Y-3rd codons.

Since UNN anticodons were already in use in all the codon boxes of Stage 1 organisms, it was expeditious for the nascent Stage 2 organisms to adopt Solution I, using post-transcriptional modification enzymes to convert UNN anticodons into *UNN. Such conversion proceeded readily in the CAN, AAN, GAN, and AGN boxes in the right half of the code that employs R-2nd codons, but not in the codon boxes in the left half of the code that employs Y-2nd codons owing to the different allowable codon-anticodon configurations in the two halves [55,56,57]. Among extant organisms, modifications of U34 to form xm^5^U34, s^2^U34, and Um34 represent the major routes to a restricted *UNN [58]. The recognition signals for these modifications are largely non-elucidated for any organism, except that the signal for ribose methylation on N34 appears to reside outside the anticodon [59].

In splitting the four codons in a 1aa codon box such as the CCN box for Pro into two pairs, one may leave the CCY codons to Pro and give the CCR codons to an NCAA, or vice versa. Giving the CCR codons to NCAA could be preferable unless the existing UGG anticodon for Pro is already in the *UGG form. This requires a search for suitable orthogonal tRNA(Ncaa)s bearing UGG and CGG anticodons. Since a UGG anticodon might misread the CCU and CCC codons, the UGG anticodon on the sought-for tRNA(Ncaa) should be conducive to being modified into a restricted *UGG by host enzymes. There is a paucity of information on recognition signals on tRNA that direct the enzymic conversion of a UNN anticodon to *UNN. More broadly, we earlier cloned the genes for *B. subtilis*, *A. fulgidus*, and bovine tRNA(Trp)s into *E. coli* host, and examined the post-transcriptional modifications on their gene products [46]. The results showed that (Table 1):

(a) All the cloned tRNA(Trp)s displayed s^4^U in resemblance to native *E. coli* tRNA(Trp) even though native *B. subtilis* and native bovine tRNA(Trp)s were devoid of this modification. Both cloned *B. subtilis* and native *E. coli* tRNA(Trp)s contained Cm, although native *B. subtilis* tRNA(Trp) lacked this modification. Native bovine tRNA(Trp) contained m^1^A and m^2^G, whereas cloned bovine and native *E. coli* tRNA(Trp)s were devoid of these modifications. These findings showed the decisive influence of host enzymes regarding some modifications on exogenous tRNAs.
(b) On the other hand, native and cloned *B. subtilus* tRNA(Trp)s both contained i^6^A, but native *E. coli* tRNA(Trp) did not. Also, native and cloned bovine tRNA(Trp)s both contained Gm, but native *E. coli* tRNA(Trp) did not. These findings showed that the sequence of an exogenous tRNA could be a more important determinant than host enzymes for other modifications.

Accordingly, when a UNN anticodon-bearing orthogonal tRNA(Ncaa) is introduced into a host cell, both the sequence of the tRNA and the specificity of host enzymes could impact on the post-transcriptional modification profile of the tRNA. As a result, there is a possibility, but no certainty, that any orthogonal tRNA(Ncaa) with a UNN anticodon would be converted to a restricted tRNA(Ncaa) with a *UNN anticodon. Therefore multiple candidate tRNA(Ncaa)s may have to be screened to find one that yields a *UNN. Insofar that the UNN anticodons for all the 2aa codon boxes of any host cell are prone to conversion to *UNN by host enzymes, the chances of a tRNA(Ncaa) attracting a similar conversion by host enzymes might be increased if its anticodon loop sequence resembles those of the UNN anticodon-bearing tRNAs for 2aa boxes in the host cell.

In the event that repeated trials fail to produce a *UGG-bearing tRNA(Ncaa) to read a CCA codon to be reassigned to an NCAA, Solution II can be adopted by retiring all CCA codons from the proteome, and deleting from the genome all tRNA(Pro)s that read the CCA codon. Although the presence of unassigned codons that are not assigned to any amino acid or termination signal could be detrimental to an organism, the extent of the detriment might not always be severe [53], and unassigned codons are also known to exist in organisms [60,61]. Moreover, when the CCA codon is entirely retired from the proteome, it becomes an absent codon rather than an unassigned codon and unable to cause damage except where it reappears through random point mutations. Such random reappearances would be tolerated by the cells as in the case of a great majority of random point mutations if they do not cause significant damage, and eliminated by natural selection if they do. Thus, either way, their perturbation of the reassignment process could be limited.

## 5. Discussion

By means of electric discharge, high-energy particle bombardment, and conveyance on meteorites, etc., prebiotic Earth was endowed with the presence of a range of different amino acids [62]. After primitive RNA genes accumulated through selection of aptamers and ribozymes over non-functional RNAs to initiate the RNA world, attachment of amino acids to these functional RNAs (fRNAs) furnished much needed extra sidechains to enhance catalytic and transporter activities. The fRNAs developed mRNA domains to encode peptide prosthetic groups composed of Phase 1 prebiotically-derived amino acids [48,63]. The sidechain imperative continued to propel the coevolution of genetic code and amino acid biosynthesis, adding biosynthetically derived Phase 2 amino acids to the code [64,65]. Even after establishment of the standard genetic code, more Phase 3 amino acid residues are added to proteins via post-translational modifications (PTM) [66]. There are more than 87,000 experimentally-identified PTMs on 530,264 proteins including phosphorylation, acetylation, glycosylation, amidation, hydroxylation, and d-amino acids, etc., such that the rate of detection of PTM sites is outpacing biological knowledge of the function of those modifications [67,68]. This flourish of PTMs underlines the sustained strength of the sidechain imperative as a factor in protein evolution. In just over three decades since the isolation of the *B. subtilis* genetic code mutants [1], a wide range of mandatory and optional synthetic lifeforms have been developed incorporating a comparable number of code mutation-induced Phase 4 NCAAs as PTM-induced Phase 3 NCAAs into proteins [1,4,14,15,16,17,20,21,22,23,24,25,26,27,28,29,30,31,32,33,37], attesting to the continuing need, now scientifically-perceived instead of evolution-driven, for more amino acid sidechains.

The methods that have been developed to produce Phase 4 protein alphabets include proteome-wide replacement of CAA by NCAA, NCAA insertion through suppression of nonsense codons, and NCAA encoding through the pyrrolysine and selenocysteine pathways. Efforts to encode NCAAs through proteome-wide reassignment of nonsense or sense codons are also ongoing. Reassignment of non-rare codons is a large-scale operation, but genome editing and synthetic genomes are powerful tools well suited to the task. Such reassignments may meet with hurdles posed by anticodons that double as tRNA identity elements and, in the event of codon box splitting, misreading of NNY codons by UNN anticodon. However, the results shown in Figure 1 and Table 1, together with the option of Solution II abandoning both the NNA codon and UNN anticodon for a split box suggest that these hurdles could be surmountable in at least some instances. Deepened insight into the structure-function relations of ARSs and tRNAs, especially archaeal ones as a rich source of orthogonal components, together with an understanding of the recognition signals on tRNA for the conversion of UNN anticodons to *UNN in different host systems would be most valuable. In addition to NCAAs that are unknown to living cells, Phase 3 NCAA residues produced by PTM likewise merit encoding so they can become Phase 4 NCAAs, freed from their hitherto strict dependence on PTM enzymes and insertable anywhere in the proteome. These PTM-generated NCAA residues have long served important functions in vivo, and their catabolic products are non-toxic at modest levels.

In the triplet genetic code, the UCN Ser, CUN Leu, and CGN Arg codon boxes can be reassigned to three NCAAs without codon box splitting. Allowing box splitting and leaving two codons to each CAA outside of Met and Trp can accommodate up to 12 NCAAs. Switching from triplet to quadruplet codons [29] gives rise to 256 codons, and addition of an extra X-Y base pair to the current A-T and G-C base pairs [11,12,13] gives rise to 216 triplet codons, both vastly increasing the number of NCAAs that might be packaged into the same code. Since different synthetic lifeforms may be allocated different sets of NCAAs, there will be ample scope to construct revised triplet codes using just the A-T and G-C base pairs, each encoding 20 CAAs, Sec, Pyl plus one or more NCAAs, for the exploration of wide ranging applications of NCAA-enhanced protein alphabets including the following:

### 5.1. Protein Structure-Activity Relationships

Active sites of enzymes are often strongly conserved in localized sequence space owing to an optimally evolved protein configuration. However, the strong conservation renders it difficult to assess the uniqueness of the evolved configuration. In this regard, when the genomes of TR7 revertants of synthetic lifeform HR23, in which Trp has regained the ability to support cell propagation, were sequenced, their RNA polymerases were found to harbor the β-Glu433Lys, β′-Ile280Thr or β′-Pro277His mutations even though the β-Glu433, β′-Ile280 and β′-Pro277 residues at the outer claw-like region of bacterial RNAP where sigma factor binds are strongly conserved [5]. Without the immense stress placed on the cells by the proteome-wide displacement of Trp by 4FTrp, it would be highly unlikely to encounter the presence of β-Lys433, β’-Thr280, or β’-His277 in an alternative active configuration of the RNAP claw. Consequently, when NCAAs are inserted into diverse positions throughout the proteome, they can reveal unforeseen insights into the structure-activity relationships of many proteins.

### 5.2. Peptide and Protein Drugs

Novel peptide/protein drugs, antibodies and industrial proteins are applications that can benefit from NCAAs. Eight NCAA residues, including d- and dl-amino acids, have been incorporated into icatibant, a bradykinin B2 receptor antagonist decapeptide [69], and d-amino acid containing peptides furnish candidate drugs to inhibit the aggregation of amyloid peptides in Alzheimer’s disease [70]. Conversion of cystine to diselenide in alpha-selenoconotoxins increases drug efficacy through prevention of reduction by glutathione or serum albumin [71]. NCAA encoding would accelerate development of such peptide or protein drugs where the presence of NCAA expands the physicochemical properties of their sidechains. Since the ∆∆G for transfer from octanol to water of 4FTrp, 6FTrp, or 5FTrp compared to Trp amounts to 0.42, 0.80, or 0.90 kcal/mole, respectively, replacement of Trp by 4FTrp, 6FTrp or 5FTrp can bring about a graded increase in hydrophobicity [72]; gamma-carboxyGlu can enhance charge intensity [73]; and irreversible attachment of the drugs to their target sites can be achieved using NCAAs capable of click chemistry [21,22,74].

MicroRNAs (miRNAs), small interfering RNAs (siRNAs), and peptide motifs such as zinc fingers, play important roles in gene regulation and represent potential therapeutic agents [75,76,77,78]. Nuclease-resistant nucleobase-peptides and nucleobase-proteins comprising both regular peptide segments and polyamide nucleic acid (PNA) [79] segments can be synthesized through the encoded incorporation of NCAAs bearing U(T), C, A, and G nucleobase sidechains. Given the capability of PNA for strong complementary base-pairing, nucleobase-peptides can provide therapeutic agents that interact with DNA advantageously with cooperation between the nucleobase sidechains and other amino acid sidechains.

### 5.3. Enhancement of Biological Fitness

The evolution of the genetic code was guided by the increased biological fitness made possible by extra amino acid sidechains in the protein alphabet, and this can be continued through expansion of the code to include NCAAs as exemplified by the finding that placement of 3-iodoTyr into the Tyr39 position of holing enhanced the competitive fitness of T7 phage [80]. Asn and Gln are highly prone to thermal deamidation [81], and the more thermostable albizzine (α-amino-β-ureidopropionic acid) has been proposed as a possible Gln replacement [82]. Since the deamidation of Asn and Gln in proteins could contribute to senescence, as well as amyloid formation in dementia, type 2 diabetes, cataracts and Parkinson’s disease [83,84,85], it would be instructive to replace Asn and Gln residues in proteins with competent yet more stable analogues, if such can be found, to assess the roles of Asn and Gln as factors of senescence and human diseases.

### 5.4. Metabolic and Biomimetic Engineering

The opening up of selenoprotein biochemistry via unconstrained proteome-wide Sec encoding enables the replacement of disulfide bridges by diselenide bridges in proteins [34,35]. As a result, metabolic engineering can be carried out replacing glutathione (GSSG) as a cellular redox buffer by selenoglutathione (GSeSeG): otherwise selenoglutathione would destabilize disulfide bridges in proteins, for the E°’ of −407 mV for GSeSeG is much more negative than that of −256 mV for GSSG [86]. The finding of 10^2^ to 10^4^-fold faster reduction of thioredoxin by synthetic seleno-glutaredoxin 3 compared to glutaredoxin 3 [87] suggests that replacement of glutathione by selenoglutathione could facilitate reductive processes such as nitrogen fixation [88,89].

The enzyme farnesyltransferase adds the 15-carbon farnesyl group to Cys in a CaaX motif at the carboxyl terminus of a protein, thereby favoring attachment of the protein to membranes. Ras proteins are activated by farnesylation and association with the inner surface of the plasma membrane [90]. Encoded incorporation of farnesylated NCAAs into various soluble proteins (if necessary using a modified EF-Tu that accommodates a bulky amino acid sidechain, or through sidechain add-on by means of click chemistry) can therefore relocate them on to membranes, thereby allowing a shift of protein distribution from cytosol to membranes. Such studies will contribute to delineation of the relationships between cellular architecture, regulation, and carcinogenesis.

Lipoproteins play important roles in metabolism and biological structures, but they are typically maintained by non-covalent rather than covalent binding between lipid and protein. However, akin to covalent nucleobase-proteins, covalent lipoproteins can be formed by incorporating lipoidal NCAAs with for example sphingosine, sphingomyelin, fatty acid, isoprenoid, or sterol type sidechains into proteins. The covalent lipoproteins can potentially be employed to produce artificial myelin sheaths and skin grafts, or circulating amphiphiles for coating the inner wall of blood vessels to prevent vascular plaques. They can also be used in a new generation of liposomes embedded with protein receptors, transporters, and ion channels as integral parts of the membrane.

An important goal in artificial photosynthesis for hydrogen fuel production is to find responsive matrices that mimic the protein matrices of natural photosynthesis, which through concerted motion can reduce entropy production and maximize the performance of essential components of the process [91,92,93,94,95]. Like natural photosynthesis, which proceeds in photosynthetic membranes where chlorophylls are held in place by specific proteins, covalent lipoprotein membranes containing ordered arrays of light harvesters, reaction centers, hydrogenase catalytic sites, etc. may provide suitable materials for engineering the necessary responsive matrices.

In conclusion, synthetic lifeforms employing rewritten genetic codes can be produced by a number of different methods. These methods will widen the scope of synthetic life research, bringing unique insights into protein chemistry and biology as well as a wide range of applications. Building the rewritten genetic codes and the novel protein alphabets ushered in by them, optimizing their uses and preventing all possibilities of misuse, will represent a momentous development that advances science, medicine and biotechnology.

## Figures and Tables

**Table 1:** Post-transcriptional modifications of tRNA(Trp)s (after [46]).

tRNA(Trp)	s^4^U	Gm	Cm	m^1^A	i^6^A	m^2^G
**Cloned *B. subtilis***	0.2	0.2	1.0	−	0.9	−
**Cloned bovine**	0.3	0.1	0.9	−	−	−
**Cloned *A. fulgidus***	0.2	0.1	−	−	−	−
**Native *E. coli***	0.5	−	1.1	−	−	−
**Native *B. subtilis***	−	−	−	−	+	−
**Native bovine**	−	+	+	+	−	+
**Native *H. volcanii***	−	−	+	−	−	−
